# Umbilical Coiling Index, Doppler Parameters, and Cord Blood Gas Analysis: Lack of Correlation in Uncomplicated Term Pregnancies

**DOI:** 10.3390/jcm15051810

**Published:** 2026-02-27

**Authors:** Zeynep Begum Celik, Gulseren Dinc, Suleyman Caner Karahan, Sumeyye Sura Ayan, Suleyman Guven

**Affiliations:** 1 Department of Obstetrics and Gynecology, Turhal State Hospital, Tokat 60300, Turkey; zeynepbegumcelik@ktu.edu.tr; 2 Department of Obstetrics and Gynecology, Faculty of Medicine, Karadeniz Technical University, Trabzon 61080, Turkey; sguven@ktu.edu.tr; 3 Department of Biochemistry, Faculty of Medicine, Karadeniz Technical University, Trabzon 61080, Turkey; suleymancanerkarahan@ktu.edu.tr (S.C.K.); sumeyyeayan@ktu.edu.tr (S.S.A.)

**Keywords:** cord blood gas, ischemia-modified albumin, MCA Doppler, umbilical coiling index, umbilical artery Doppler

## Abstract

**Background/Objectives**: In this study, we aimed to evaluate whether neonatal ischemia-modified albumin (IMA) and umbilical venous cord blood gas parameters are associated with antenatal markers of fetal well-being, including the umbilical coiling index (UCI) and umbilical artery (UA) and middle cerebral artery (MCA) Doppler indices. **Methods**: For this prospective observational study, sixty-five low-risk term pregnancies (≥37 weeks) were included. Prenatal ultrasound was used to measure the UCI and UA/MCA Doppler indices. At delivery, umbilical venous cord blood gas and serum IMA analyses were performed. Maternal and neonatal data (birth weight, 5 min Apgar score, NICU admission, sex, and delivery mode) were recorded, and correlations and group comparisons were performed (*p* < 0.05). **Results**: The UCI ranged from 0.210 to 0.471 coil/cm (mean 0.337). The UA and MCA Doppler indices were within the reference ranges. The UCI showed no significant correlation with umbilical venous blood gas values, IMA, UA/MCA Doppler indices, gestational age/weeks, or 5 min Apgar score. The UA S/D ratio and UA resistive index (RI) were negatively correlated with birth weight (*p* < 0.05). Umbilical venous pH was positively correlated with the 5 min Apgar score, whereas venous pCO_2_ was negatively correlated with the 5 min Apgar score (both *p* < 0.05). Newborns with venous pH < 7.32 had higher cesarean delivery rates and higher rooming-in rates. Newborns admitted to the NICU had higher mean UA systolic velocity/diastolic velocity (S/D) and UA pulsatility index (PI) and lower venous pH. **Conclusions**: In low-risk term pregnancies, the UCI was not associated with cord blood gas parameters, IMA, or UA/MCA Doppler indices. These results suggest that the UCI may have limited clinical utility as a predictor of early neonatal acidosis or oxidative stress in a strictly low-risk population.

## 1. Introduction

The placenta is a unique organ that supports fetal growth by providing the nutrients and substrates required for intrauterine survival and development. It is a major endocrine organ that secretes more than one hundred peptide and steroid hormones [1]. Maternal–fetal transport occurs via the umbilical cord, which typically contains two arteries and one vein; therefore, any pathology affecting the placenta or umbilical cord may compromise fetal development, including fetal cardiac activity [2].

Blood transport in fetal circulation somewhat differs from that in the postnatal period due to the intrauterine environment. A simple description of fetal circulation is as follows: Oxygenated blood from the placenta reaches the fetus via the umbilical vein. A small portion of this blood is transferred to the portal system, while the remainder passes into the ductus venosus and inferior vena cava. This blood merges with deoxygenated blood from the superior vena cava and enters the right atrium. Some of the blood passes through the foramen ovale into the left atrium, enters the aorta, and supplies the aortic arch and major vessels. The remaining blood enters the right ventricle, mostly passing into the pulmonary artery, which then supplies the remaining aorta and its branches. Finally, the blood returns to the placenta via the two umbilical arteries. Although called umbilical arteries, they carry blood that is poor in oxygen and contains cellular waste products [3].

A key structural feature of the umbilical cord is its spiral “coiling” pattern, which contributes to both flexibility and mechanical strength, protecting the cord from external forces. Coils may be oriented to the right or left, with left-sided coiling reported to be more frequent, although the determinants of coiling direction remain unclear [4]. Clinical interest in quantifying this morphology has led to the development of the umbilical coiling index (UCI) [5]. Early efforts to quantify coiling date back to Edmonds (1954), who described a “torsion index” as the ratio of cord twists to cord length [6]. Strong (1994) later simplified and standardized the concept by defining the UCI as the number of coils per unit cord length, irrespective of coiling direction [7]. In uncomplicated pregnancies, UCI distribution and reference values have been reported (mean around 0.17 coil/cm with corresponding percentile ranges), and Strong’s approach is currently accepted as the standard method [8].

**Figure 1:**
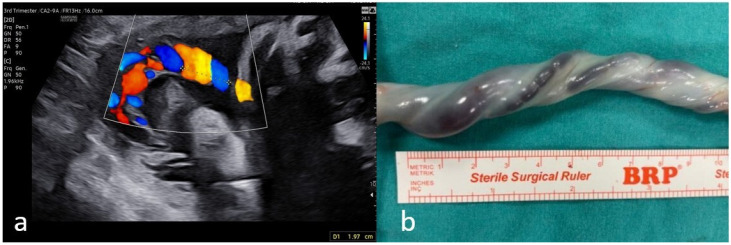
Sonographic (**a**) and postnatal (**b**) views of umbilical coiling index. The UCI was calculated as 1/1.97 = 0.507 for the sonographic image and 1/5.7 = 0.182 for the postnatal image.

The UCI can be assessed both antenatally and postnatally. Antenatal ultrasonographic assessment may be performed by dividing “1” by the distance required for a 360-degree umbilical arterial turn, while postnatal assessment may be performed by dividing the total number of coils by the total cord length [9]. Antenatal measurements obtained by ultrasound have been reported to correlate with postnatal placental measurements, although the antenatal UCI may appear higher, potentially due to segment selection and the inability to fully sample the fetal end of the cord [10]. Against this methodological background, multiple studies have investigated whether the UCI is associated with fetal problems and adverse perinatal outcomes [11,12,13]. Sonographic and postnatal views of the UCI are provided in Figure 1.

**Figure 2:**
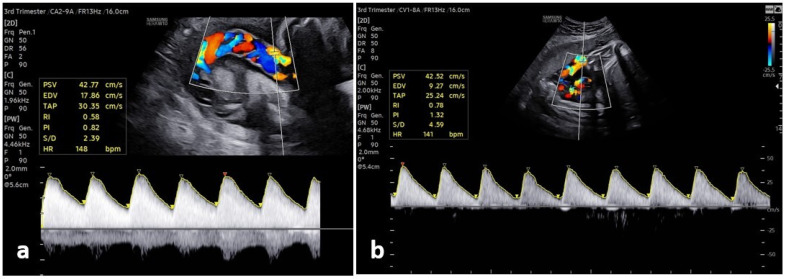
Sonographic images of fetal umbilical artery Doppler measurement in (**a**) low-risk and (**b**) high-risk cases. In the image in (**b**), the increases in S/D (systolic/end-diastolic flow ratio) and end diastole flow compared with (**a**) are noteworthy.

In routine obstetric care, Doppler assessment of the umbilical artery and middle cerebral artery has long been used to monitor fetal well-being during the antenatal period [14]. Umbilical artery Doppler examination is commonly used in IUGR (intrauterine growth restriction). In IUGR, the fetus fails to achieve normal development due to infection, genetic factors, or placental insufficiency. These factors can also affect fetal health. Small-for-gestational-age (SGA) fetuses are small in measurement but still remain healthy. Placental resistance-related insufficiency can be identified via umbilical artery Doppler examination. Depending on the degree of insufficiency, the diastolic portion of the flow wave may be reduced, absent, or reversed. Abnormal Doppler findings have been associated with poor perinatal outcomes. Some studies have suggested that if the umbilical artery Doppler is normal in SGA fetuses, a follow-up procedure similar to that used in low-risk pregnancies may be appropriate. It has long been thought that umbilical artery Doppler measurements may be early indicators of fetal hypoxemia and metabolic acidosis. In high-risk pregnancies, Doppler ultrasound evaluation has been shown to reduce maternal hospitalizations, labor induction, cesarean section due to uncertain fetal status, and perinatal mortality [15,16]. Figure 2 depicts sonographic images of fetal umbilical artery Doppler measurement in low-risk and high-risk cases.

**Figure 3:**
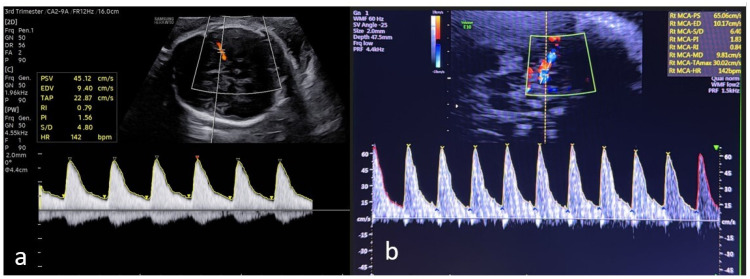
Sonographic images of fetal middle cerebral artery Doppler measurement in (**a**) low-risk and (**b**) high-risk cases. In the image in (**b**), the increase in PS (peak systolic velocity) flow compared with (**a**) is noteworthy.

Another Doppler measurement used to assess antenatal fetal well-being is performed via the middle cerebral artery (MCA). When the fetus enters hypoxia, a cerebral protective mechanism is activated, increasing blood flow to the brain and causing vasodilation in the cerebral vessels. This vasodilation results in a decrease in Doppler indices. The ratio of the MCA and UA indices (CPR, cerebroplacental ratio) has been reported to be more effective for assessing poor perinatal outcomes [15]. Generally, percentile tables based on gestational age, as described in the literature, are used to identify high-risk groups. However, these tables do not take into account some maternal factors. It has been indicated that these maternal factors should also be considered when determining risk to more effectively prevent pregnancy complications such as preeclampsia, perinatal death, and cesarean delivery due to unsafe fetal conditions. The importance of UA-PI, MCA-PI, and CPR measurements in preventing these complications has been emphasized [17]. Also, MCA-PSV (MCA-peak systolic velocity) measurement is now considered a standard in the diagnosis of fetal anemia. It has been reported that, to prevent unnecessary tests and monitoring, only pregnant women considered to be at risk of fetal anemia should undergo MCA-PSV measurement [18]. Figure 3 shows sonographic images of fetal middle cerebral artery Doppler measurement in low-risk and high-risk cases.

In parallel, biochemical markers used to assess ischemia–hypoxia have gained increased attention. Ischemia-modified albumin (IMA) arises when ischemia-related cellular changes reduce albumin’s binding capacity for certain molecules; accordingly, IMA levels are frequently examined in studies focusing on ischemia–hypoxia states [19]. In the context of newborns, IMA may also be considered a marker related to oxidative stress, while umbilical venous blood gas analysis represents one of the earliest postpartum indicators of fetal/neonatal well-being [20,21].

Based on the fundamental information provided above, this study aims to investigate whether the UCI plays an important role in the process of oxygen-rich blood reaching the fetus via the umbilical vein from the placenta. We set out to perform fetal Doppler measurements (UA and MCA) to demonstrate the effectiveness of UCI measurement on the fetal side. Similarly, to differentiate between placental ischemia and to more accurately determine the relationship between fetal UCI and Doppler parameters, the level of ischemia-modified albumin (IMA) was measured in umbilical vein serum (oxygen-rich blood samples from the placenta) to rule out placental ischemia. A comprehensive and detailed review of the literature revealed that no studies have examined the importance of factors including the UCI, fetal Doppler, and umbilical vein IMA in antenatal assessment. Therefore, we believe that our research can contribute to the literature by filling the gap in this area. One limitation of our study was that, due to the limited number of cases and the very close UCI values, we were unable to perform a grouping analysis. Future studies may avoid this limiting factor by using larger samples including high- and low-risk pregnant cases.

The present study aimed to determine whether there is a meaningful relationship among the umbilical coiling index, umbilical and middle cerebral artery Doppler parameters, umbilical venous blood gas values, and IMA measurements. Demonstrating such an association was considered potentially useful for refining antenatal surveillance strategies in fetuses evaluated as being at risk of hypoxemia, informing obstetric management and delivery-timing decisions, and enabling the planning of early postpartum neonatal care whilst still within the prenatal period.

## 2. Materials and Methods

### 2.1. Study Design

This prospective observational study evaluated term pregnancies followed in the Department of Obstetrics and Gynecology outpatient clinic and subsequently admitted for delivery. The study protocol was reviewed and approved by the Karadeniz Technical University Clinical Research Ethics Committee (meeting date: 7 November 2022; decision no: 2022/231, no: 20). This study consisted of 65 pregnant women who volunteered to participate. The study comprised women who applied to the antenatal clinic of the Department of Obstetrics and Gynecology, Karadeniz Technical University Faculty of Medicine for prenatal follow-up between 2022 and 2023; underwent vaginal delivery or cesarean section while hospitalized; and had term pregnancies (37 weeks or longer). Prenatal fetal umbilical artery and middle cerebral artery Doppler indices were measured and recorded using an ultrasonography device in our department. The ultrasound examination was performed by GD using a Philips Clear Vue 350 ultrasound device (Koninklijke Philips company, Amsterdam, The Netherland) with an abdominal ultrasound probe operating at a frequency of 2–5 MHz. Umbilical artery systolic velocity/diastolic velocity (S/D), pulsatility index (PI), and resistive index (RI) values, as well as middle cerebral artery S/D, PI, and RI values, were recorded during the ultrasound examination.

The exclusion criteria comprised multiple gestation, fetal single umbilical artery, fetal anomaly, fetal growth restriction, fetal aneuploidy, maternal gestational/pregestational diabetes, chronic hypertension, preeclampsia, or HELLP syndrome.

### 2.2. Umbilical Coiling Index and Doppler US Measurements

In this study, as previously described in the literature, the umbilical coiling index (UCI) was assessed antenatally as the reciprocal of the distance (cm) corresponding to a complete 360° turn (UCI = 1/distance in cm) [7]. The umbilical artery Doppler (S/D, PI, RI) measurement technique that we used is detailed as follows:

Umbilical artery (UA) Doppler velocimetry was performed using a pulsed-wave spectral Doppler. Recordings were obtained during fetal quiescence (absence of fetal breathing and gross body movements) using color flow mapping when the vessel and flow direction needed to be identified correctly. For consistency, the Doppler sample volume was placed on a free-floating loop of the umbilical cord, acknowledging that Doppler indices vary along the cord length (fetal end vs. free loop vs. placental end); site-specific reference ranges should be used when alternative fixed sampling sites are required. The Doppler settings were optimized to obtain a clear maximum velocity envelope (appropriate PRF/scale and sweep speed), and the vessel wall (high-pass) filter was kept low to avoid artifactual reduction/absence of end-diastolic flow. At least three consecutive uniform waveforms were recorded, and the ultrasound system-derived indices were documented: S/D ratio = PSV/EDV; RI = (PSV − EDV)/PSV; and PI = (PSV − EDV)/TAV (where PSV is peak systolic velocity, EDV is end-diastolic velocity, and TAV is time-averaged velocity) [22,23].

### 2.3. Middle Cerebral Artery Doppler (S/D, PI, RI) Measurement Technique

Middle cerebral artery (MCA) Doppler was obtained on an axial plane of the fetal head, including the thalami and sphenoid bone wings. Color flow mapping was used to visualize the circle of Willis and identify the proximal MCA. The pulsed-wave Doppler gate was positioned at the proximal third of the MCA, close to its origin from the internal carotid artery. The insonation angle was kept as close to 0° as possible, and unnecessary pressure on the fetal head was avoided. At least three, but fewer than ten, consecutive waveforms were recorded under fetal rest, and MCA Doppler indices (S/D, RI, PI) were calculated from the traced spectral envelope using the same standard definitions (with PI commonly obtained via autotrace when available) [22].

Umbilical venous blood gas and biochemical sampling. During delivery, umbilical venous blood gas and serum samples were obtained. For IMA analysis, blood was collected in a 5 mL serum separator gel biochemistry tube immediately before placental separation from the uterus. For blood gas analysis, blood was drawn from the umbilical vein into a heparinized syringe and transported to the biochemistry laboratory. After clotting, serum was centrifuged at 1200× *g* and stored at −20 °C until the IMA measurement was made. Umbilical venous blood gas analyses were performed using a Radiometer ABL800 gas analyzer device (Radiometer medical device company, Istanbul, Turkey); pH, pO_2_, and pCO_2_ values were recorded.

### 2.4. Ischemia-Modified Albumin (IMA) Measurement

To determine IMA levels, the albumin cobalt binding test was used, and the decreasing binding capacity of cobalt to albumin was evaluated using the colorimetric determination method developed by Bar-Or et al. [24]. A total of 200 μL of serum sample was added to plastic cuvettes. Then, 50 μL of 0.1% CoCl_2_ · _6_H_2_O was added and the cuvettes were gently shaken. The sample was left for 10 min to allow for sufficient cobalt binding to the albumin, after which 50 μL of 1.5 mg/mL Dithiothreitol (DTT) was added as a coloring agent. After waiting for 2 min, 1 mL of 0.9% NaCl was added to stop the reaction. A blank was prepared for each sample. In the step where DTT was added, 50 μL of distilled water was added instead of 50 μL of 1.5 mg/mL DTT to prepare a DTT-free serum cobalt blank. Sample absorbances were measured and recorded at a wavelength of 470 nm using a spectrophotometer. Color formation in DTT samples was compared with color formation in blank tubes, and the results were given in absorbance units (ABSU). Serum albumin levels were measured using the Bromocresol Green (BCG) photometric method on a Beckman Coulter AU 5800 (Beckman Coulter Inc., Brea City, CA, USA) autoanalyzer. The serum albumin results were found to be an average of 32.50 ± 2.47 g/L. To minimize the potential influence of variations in the serum albumin concentration on the IMA values, the ischemia-modified albumin/albumin ratio (IMAR) was calculated as IMAR = IMA (ABSU)/albumin (g/L) [24,25].

Clinical and neonatal variables: Maternal age, gravida, parity, gestational week at delivery, neonatal birth weight, 5 min Apgar score, mode of delivery, neonatal intensive care unit admission status, and neonatal sex were recorded.

### 2.5. Outcomes

The primary outcome of this study was the relationship among the UCI, umbilical and middle cerebral artery Doppler indices, umbilical venous blood gas parameters (pH, pO_2_, pCO_2_), and ischemia-modified albumin (IMA). The recorded maternal/neonatal characteristics (e.g., birth weight, 5 min Apgar, delivery mode, NICU admission) were evaluated as additional clinical variables.

### 2.6. Statistics

Descriptive statistics were reported as numbers and percentages for categorical variables and means ± standard deviations for continuous variables. Normality was assessed using the Kolmogorov–Smirnov test. Pearson’s correlation analysis was used to evaluate linear associations, and Student’s *t*-test was used to compare means. Statistical significance was set at *p* < 0.05, and analyses were performed using IBM SPSS Statistics 11.5 (SPSS company, Chicago, IL, USA).

The G*Power 3.1.9.7 program was used for sample size and power analysis. Based on the results of a preliminary study, when the effect size was set at 0.80 and 22 subjects were included in each group (NICU requirement and rooming-in groups), the alpha value was 0.05 and the power was 0.83.

## 3. Results

A total of 65 term pregnancies were included in this study; the maternal age of the participants ranged from 19 to 39 years. The maternal and neonatal demographic characteristics of the study group are given in Table 1.

The mean umbilical coiling index (UCI) was 0.33 ± 0.06 coil/cm (range, 0.21–0.47), and the ischemia-modified albumin/albumin ratio (IMAR) in cord blood ranged from 0.03 to 0.05 (mean 0.04 ± 0.007) for the whole study group. Umbilical artery and middle cerebral artery Doppler parameters were not observed outside of the ranges referenced in the literature in this cohort.

No statistically significant correlation was identified between the UCI and umbilical venous blood gas parameters, IMAR, umbilical artery Doppler indices, middle cerebral artery Doppler indices, maternal age, gestational week, or 5 min Apgar score. Significant correlations are outlined in Table 2.

Threshold analysis for umbilical venous pH: When the umbilical venous pH was <7.32, the cesarean delivery rate was higher (80.9%), and the proportion of newborns that roomed-in with their mother was 52.38%.

No significant differences were found between the groups for the UCI, MCA Doppler parameters, other umbilical artery Doppler parameters (except PI), or IMA/IMAR measures (Table 3).

Among NICU admissions, the most frequently recorded clinical indication was grunting (59.1%), followed by severe respiratory distress requiring intubation (22.7%); pneumonia, wet lung, delivery-related complication, and early-onset sepsis were each reported at 4.5%.

**Figure 4:**
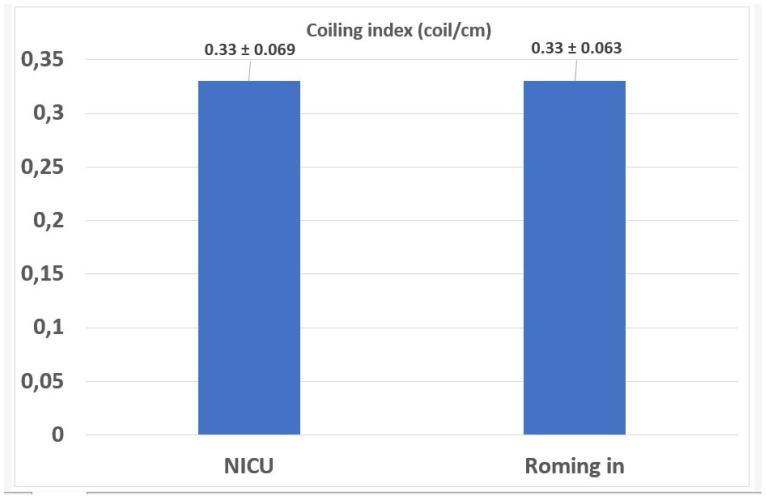
Comparison of mean ± standard deviation UCI values in NICU admission and rooming-in groups (*p* = 0.89, Student’s *t*-test was used for comparison).

A comparison of the mean ± standard deviation UCI values in the NICU admission and rooming-in groups is provided in Figure 4.

## 4. Discussion

The present study evaluated whether the umbilical coiling index (UCI) is meaningfully associated with antenatal fetal well-being markers (umbilical artery and middle cerebral artery Doppler indices) and early postnatal hypoxemia/ischemia-related indicators (umbilical venous blood gas parameters and ischemia-modified albumin, IMA/IMAR) in a cohort of uncomplicated term pregnancies. As a quantitative descriptor of umbilical cord twisting (coils/cm), the UCI can be assessed antenatally via ultrasound and interpreted against the values reported for an uncomplicated pregnancy [7,8,9]. The principal finding of this study was that the UCI showed no significant correlation with umbilical venous blood gas values, IMA/IMAR, or UA/MCA Doppler indices. However, several “secondary” relationships did emerge that were clinically coherent: umbilical artery S/D and RI were inversely correlated with birth weight, and umbilical venous pH correlated positively with 5 min Apgar, while pCO_2_ correlated negatively. Additionally, newborns requiring NICU follow-up demonstrated higher UA S/D and PI and lower umbilical venous pH than those in the rooming-in group, whereas the UCI and IMA/IMAR did not differ between these groups. It is noteworthy that while the UCI and IMA/IMAR values were similar between the NICU and rooming-in groups, standard physiological parameters—specifically UA Doppler resistance indices and venous pH—effectively differentiated these neonates. This reinforces the fact that even in a low-risk population where structural markers like the UCI may lack discriminatory power, hemodynamic and metabolic indicators remain sensitive markers of neonatal clinical status.

A key interpretive point is the clinical context and distribution of the UCI in the current sample. Our cohort was restricted to term pregnancies (≥37 weeks) without major maternal/fetal pathology, which likely narrowed down the range of placental resistance and fetal compensatory responses [1,2]. The mean UCI was 0.33 ± 0.06 (0.21–0.47); this range is broadly consistent with the values reported for an uncomplicated pregnancy [8], and the participants in this study did not show classic “high-risk” indications such as fetal growth restriction, preeclampsia, diabetes, or congenital anomalies. In such a low-risk setting, the UCI may not vary sufficiently or may not reflect a clinically meaningful compromise to translate into detectable differences in Doppler indices or biochemical markers.

The methodology of this study is also relevant. Antenatal Doppler sampling was standardized to a free-floating loop of the cord, with explicit acknowledgment that Doppler indices can vary by sampling site along the cord (fetal end vs. free loop vs. placental end). Similarly, antenatal UCI estimation may be influenced by segment selection and by the practical limitation that not all segments of the cord (particularly the fetal end) can be fully sampled in every pregnancy. These factors can dilute the associations made across studies and can partly explain why some cohorts report stronger relationships between the UCI and fetal compromise than others. This finding aligns with studies that have placed prior emphasis on detailed cord evaluation in prenatal ultrasound and the challenges of standardized segment sampling [5,9].

Previous work has proposed that abnormal coiling patterns (hypocoiling/hypercoiling) may be linked to adverse perinatal outcomes, including mechanisms related to altered cord flexibility and vulnerability to compression or hemodynamic changes (e.g., studies that examine UCI categories and perinatal endpoints). Spiral twisting is a normal anatomical feature of the umbilical cord, and variations in cord structure and coiling have long been described in the anatomic and obstetric literature [4,10]. Some researchers have reported associations between the UCI and perinatal outcomes [10,14,26,27]. However, a consistent theme across the literature is heterogeneity in (i) how the UCI is measured (antenatal vs. postnatal, which segment); (ii) the clinical risk profile of the cohort; and (iii) the endpoints used (growth restriction, intrapartum compromise, acidosis, NICU admission). Therefore, the absence of a relationship in a rigorously “uncomplicated term” cohort should not be interpreted as evidence against the UCI’s potential relevance in higher-risk settings; rather, this suggests that the UCI may have limited discriminatory value when the baseline risk and physiologic variability are low.

Regarding the UCI and Doppler, some studies have explored whether the antenatal UCI correlates with Doppler flow characteristics [13,22,28,29]. However, various datasets have not shown a stable relationship between Doppler indices and fetal acid–base status across contexts (e.g., mixed or special populations), highlighting that the UCI–Doppler–acidemia pathway, if present, may be conditional on the presence of placental/cord pathology or clinically meaningful hypoxemia. In our cohort, the UA/MCA Doppler values remained within the expected reference ranges overall [23,30], which again reduces the likelihood of detecting UCI-related hemodynamic signatures.

Although the UCI was not associated with Doppler metrics in our study, UA Doppler indices still carried biologically plausible information in this cohort. The observed inverse correlation between UA S/D and RI with birth weight aligns with the concept that increased placental vascular resistance—captured even within a “normal” range—can be associated with lower fetal growth potential. Prior studies have similarly linked UA Doppler patterns to birth weight and adverse outcomes in specific clinical settings [31,32,33,34]. This finding supports the continued role of UA Doppler as an antenatal surveillance tool, even when structural cord parameters such as the UCI do not add explanatory value in low-risk term pregnancies. Although the UA Doppler indices in this study were generally within normal reference ranges, the significantly higher S/D and PI values observed in NICU-admitted newborns suggest that subtle increases in placental resistance may still carry clinical implications for immediate neonatal transition, a distinction that was not captured by the UCI.

The correlations between umbilical venous pH and 5 min Apgar (positive) and pCO_2_ and 5 min Apgar (negative), together with the finding of lower venous pH in NICU-admitted newborns, indicate that early acid–base status tracked neonatal clinical condition in this cohort. This is further supported by the NICU indications, where respiratory distress-related problems were prominent (e.g., grunting and severe respiratory distress requiring intubation). Collectively, these results suggest that—within the constraints of venous sampling—cord blood gas parameters can still function as meaningful early postnatal signals of physiologic adaptation.

At the same time, interpretation should explicitly acknowledge well-described arterial vs. venous differences and timing effects. Umbilical cord blood gas reflects the acid–base status up to the time of clamping, but ongoing metabolic changes in the clamped segment can shift values over time; changes are minimal in the first minute, but they can become more significant with prolonged delays. Importantly, arterial blood more directly reflects fetal acid–base status, and a normal venous sample does not necessarily exclude arterial acidosis; large artery–vein differences can occur, particularly in acute events [24,25]. Therefore, the absence of strong associations between the UCI and “hypoxia markers” in this study should be viewed in light of (i) our low-risk sample and (ii) the use of venous-only cord blood gas measurements.

IMA has been proposed as a biochemical marker reflecting ischemia/hypoxia-related modification of albumin binding capacity, and in the obstetric/neonatal context, it has been discussed as an oxidative stress-related indicator. Prior work summarized in this thesis indicates that increased umbilical cord IMA levels may reflect intrauterine hypoxia or birth asphyxia and that IMA can rise rapidly after ischemic events [24,25,32]. However, in the present cohort, IMA/IMAR did not correlate with the UCI, Doppler indices, or venous blood gas parameters, and it did not differ between the NICU and rooming-in groups, despite the fact that there were measurable differences in UA S/D, UA PI, and venous pH.

Several explanations can be given for our findings that are consistent with this study’s design. First, the cohort intentionally excluded major pathologies that might generate a strong oxidative stress phenotype (e.g., growth restriction, hypertensive disease), likely producing a narrow IMAR distribution. Second, IMA/IMAR may be more informative in clearly hypoxemic/ischemic subgroups or specific complications, whereas mild physiologic variation at term may not produce a detectable biochemical separation. Third, because cord blood gas and IMA reflect different biological domains (acid–base balance vs. ischemia/oxidative modification), discordance between these signals is possible in a low-risk term population where NICU admission can be driven by postnatal respiratory adaptation rather than chronic intrauterine compromise.

This study had a high cesarean rate. In this manuscript, we discuss prior studies that report no significant difference in cord pH between vaginal and elective cesarean deliveries in some cohorts (with differences in pO_2_), and a higher NICU admission/oxygen requirement after elective cesarean in other settings [35,36]. While delivery mode differences could influence neonatal transition and NICU utilization, the current dataset’s limited number of vaginal deliveries restricts our ability to make robust comparisons; nonetheless, it underlines why cohort composition can substantially affect the observed relationships among the UCI, Doppler indices, and postnatal parameters. Delivery method can potentially confound results. However, it may also enable a less affected, more homogeneous group of individuals to be obtained that is not exposed to the stress and lengthy process of normal vaginal delivery compared to that of cesarean section. In this way, delivery method may have yielded important results in terms of the effect of the UCI alone.

Our study has several limitations. The most important of these is the small sample size (22 cases in the NICU group) and the power of 0.83. Secondly, due to our status as a tertiary hospital, we included cases with a high cesarean section rate to provide clear results for low-risk cases. Thirdly, only Doppler ultrasound was used to evaluate the effect of the ICU on fetal well-being parameters; umbilical artery blood gas analysis would have been more valuable in this context. Arterial sampling better reflects fetal acid–base status, but venous normality does not exclude arterial acidosis, and acid–base parameters may shift with sampling delays [21,36]. However, for umbilical vein blood gas analysis, umbilical vein blood IMA level measurement may have also been useful to support the accuracy of the relationship between the UCI and Doppler parameters.

## 5. Conclusions

In uncomplicated term pregnancies, the UCI was not meaningfully associated with fetal UA/MCA Doppler indices. However, UA Doppler indices (S/D and RI) were inversely related to birth weight, and neonatal condition aligned with acid–base status. Since our study did not include high-risk pregnancies, further research is needed to understand the effect of UCI measurement in these cases. In summary, our findings show that the UCI may be a useful method for monitoring uncomplicated low-risk pregnancies, but larger-scale studies are needed to determine its effectiveness.

## Figures and Tables

**Table 1:** Maternal and neonatal demographic data of the study group.

**Maternal Demographics**	
Age (years) ^a^	30.1 ± 4.7
Gestational age (weeks) ^b^	38 weeks 6 days
Gravidity ^b^	2
Parity ^b^	1
Nulliparity (%) ^c^	44.6% (29)
Cesarean delivery (%) ^c^	87.7% (57)
**Neonatal Demographics**	
Fetal male gender (%) ^c^	47.6% (31)
5 min Apgar score ^a^	8 ± 1
Mean birth weight (g) ^a^	3314.11 ± 435.02
NICU requirement (%) ^c^	33.8% (22)
Umbilical venous pH ^a^ Umbilical venous pO_2_ ^a^ Umbilical venous pCO_2_ ^a^	7.3 ± 0.05 28.34 ± 8.36 44.19 ± 7.36

NICU, neonatal intensive care unit. ^a^ Mean ± standard deviation or ^b^ median or ^c^ percentages and number of cases in parentheses are given for comparison.

**Table 2:** Correlation analysis results of study parameters.

Variables	r	*p*-Value
Umbilical artery S/D vs. birth weight	−0.34	<0.05
Umbilical artery RI vs. birth weight	−0.30	<0.05
Umbilical venous pH vs. 5 min Apgar	0.38	<0.05
Umbilical venous pCO_2_ vs. 5 min Apgar	−0.37	<0.05

RI, resistance index; S/D, systolic/diastolic ratio. Pearson’s correlation test was used for the statistical calculation.

**Table 3:** Comparison of selected parameters in terms of postnatal follow-up (NICU vs. rooming-in).

Parameter	NICU (*n* = 22)	Rooming-In (*n* = 43)	*p*-Value
Umbilical artery S/D	2.43 ± 0.51	2.15 ± 0.43	**0.02**
Umbilical artery PI	1.00 ± 0.32	0.85 ± 0.23	**0.03**
Umbilical venous pH	7.31 ± 0.06	7.34 ± 0.03	**0.01**
IMAR	0.044 ± 0.008	0.042 ± 0.007	0.40

Abbreviations: PI, Pulsatility index; S/D, systolic/end-diastolic ratio; IMAR, ischemia-modified albumin/albumin ratio. Mean ± standard deviation is given for comparison. Student’s *t*-test was used for comparison. ± indicates standard deviation. Bold values indicate statistical significance.

## Data Availability

All data are available upon request from the corresponding author.

## Abbreviations

The following abbreviations are used in this manuscript:

UCI	Umbilical coiling index
SGA	Small for gestational age
IUGR	Intrauterine growth restriction
IMA	Ischemia-modified albumin
UA	Umbilical artery
MCA	Middle cerebral artery
NICU	Neonatal intensive care unit
PI	Pulsatility index
RI	Resistivity index
S/D	Systolic/end-diastolic ratio
IMAR	Ischemia-modified albumin/albumin ratio
